# Assessing patient-level risk factors for evidence-based early diagnosis of maternal sepsis

**DOI:** 10.1186/s12884-025-07895-4

**Published:** 2025-07-22

**Authors:** Philip Emeka Anyanwu, Paul Expert, Kate Honeyford, Oluwasomidoyin Bello, Mobolaji Modinat Salawu, Ikeola Adeoye, Ayo Stephen Adebowale, Amen-Patrick Nwosu, Summia Zaher, Peter Ghazal, Adeniyi Francis Fagbamigbe, Magbagbeola David Dairo, Ceire Costelloe

**Affiliations:** 1 Warwick Applied Health, Warwick Medical School, University of Warwick, Coventry, UK; 2 Global Digital Health Unit, School of Public Health, Imperial College London, London, UK; 3 Global Business School for Health, Faculty of Population Health Sciences, University College London, London, UK; 4 Health Informatics team, Division of Clinical Studies, Institute of Cancer Research, London, UK; 5 Department of Obstetrics and Gynaecology, College of Medicine, University of Ibadan, Ibadan, Nigeria; 6 Department of Epidemiology and Medical Statistics, College of Medicine, University of Ibadan, Ibadan, Nigeria; 7 Department of Obstetrics and Gynaecology, Cardiff and Vale University Health Board, Heath Park, Cardiff, UK; 8 Systems Immunity Research Institute, Division of Infection and Immunity, Cardiff University, Cardiff, UK

## Abstract

**Background:**

Maternal sepsis is a leading cause of maternal death, with the burden higher in low- and middle-income countries (LMICs). Early Warning Systems (EWS) combine clinical observations to identify a pattern consistent with an increased risk of clinical deterioration and have been introduced for monitoring sepsis risk. Maternal sepsis risks in LMICs are driven by factors at the health system and patient levels. This study assessed patient-level risk factors -age, health-seeking behaviour, comorbidities and procedures- associated with maternal sepsis in an urban tertiary hospital in Nigeria.

**Methods:**

We conducted a retrospective study using health records of 4,510 patients from obstetrics and gynaecology units at a tertiary hospital in southwestern Nigeria from 2016 to 2020. To examine the association between patient-level risk factors and sepsis, we analysed data for the 565 maternal patients with a record of infection using a multiple logistic regression model. We extended the model by introducing interaction terms to assess whether the association between the risk factors and maternal sepsis varied by socio-demographic factors.

**Results:**

About one-fifth of the 565 maternal patients with an infection had sepsis. Patients with sepsis had the lowest rate of live birth (29.7%) compared to those with (41.8%) and without (82.1%) an infection. Proportions of stillbirth (intrauterine fetal death) and early neonatal deaths were highest among patients with sepsis (15.3% and 1.8%) compared to those with (13.2% and 2.1%) and without (4.5% and 1.7%) an infection. Antenatal care booking status (OR: 0.17; 95% CI: 0.08–0.38) and having a catheter (OR: 2.60; 95% CI: 1.35–5.01) were significantly associated with maternal sepsis in the adjusted model.

**Conclusion:**

Our results suggest that improving access to antenatal care services for pregnant women will substantially reduce the risk of maternal sepsis in the Nigerian population. Guidelines for maternal sepsis management should consider subgroups of patients at higher risk, such as those with urethral catheters.

## Introduction

The global maternal mortality rate due to maternal sepsis and infections was estimated at 12.44 deaths per 100,000 livebirths (i.e. about 16,840 deaths) in 2019; about 4 in 5 of these deaths occurred in Low- and Middle-Income countries (LMICs) [1]. Maternal infection can seriously affect the health of women and neonates. During pregnancy, acute maternal infections can cause maternal morbidity and mortality and obstetric complications such as stillbirth, miscarriage, low birth weight and preterm labour [2]. Annually, one million neonatal deaths are attributed to maternal infections or sepsis [3–6], which are preventable and treatable causes of maternal morbidity and mortality. However, according to findings from the WHO Global Maternal Sepsis Study Research Group, in 2017, around 70 out of every 1,000 live births involved pregnant or recently pregnant women requiring hospital care due to maternal infections; also, infections were associated with over half of the intrahospital maternal deaths in the same year [7]. Despite the disproportionate burden of maternal sepsis in LMICs, the evidence base for its early detection remains limited [8]. Gaps persist in understanding patient-level risk factors, such as health-seeking behaviour, and evaluating diagnostic and outcome-related parameters within LMIC settings.

According to the WHO, maternal sepsis is a life-threatening condition defined as organ dysfunction resulting from infection during pregnancy, childbirth, post-abortion, or postpartum period [7]. Escherichia coli is consistently identified as the most common causative organism in cases of maternal sepsis; other frequently reported pathogens, in descending order of prevalence, include Group B Streptococcus, Staphylococcus aureus, Group A, C, and G Streptococcus, Enterobacter species, and Chlamydia trachomatis [9–12]. However, the relative frequency of these organisms may vary across different populations and clinical contexts, such as the mode of delivery or geographic setting. Some systemic infections are also more severe during pregnancy (e.g. malaria, tuberculosis, influenza, herpes) [13].

Physiological, immunological and mechanical changes in pregnancy predispose women to infection (particularly urogenital and healthcare-associated infections, as well as other non-reproductive infections, e.g. pneumonia), placing them women at a ten-fold higher risk of developing sepsis [14]. Physiological adaptations to pregnancy can obscure early signs of infection and sepsis, delaying diagnosis and increasing the risk of rapid progression to septic shock, with severe consequences for both mother and fetus. In LMICs, this diagnostic uncertainty often leads to the inappropriate use of antibiotics, contributing to antimicrobial resistance (AMR) [15, 16].

Empirical antibiotic use and AMR complicate the already challenging task of diagnosing sepsis, particularly in resource-limited settings. Increasing resistance to first-line antibiotics, especially in LMICs, as demonstrated in a global meta-analysis [17], reduces the effectiveness of standard treatments and limits therapeutic options [18]. Previous antibiotic exposure has been shown to double the AMR risk for common respiratory and urinary tract infections [17], further complicating early and accurate identification of sepsis. These dynamics highlight the critical need for sepsis diagnostics with better specificity and negative predictive value to inform more prudent antibiotic use and improved outcomes in maternal care.

### M-EWS in LMIC/Nigeria

Early Warning Systems (EWS), incorporating clinical observation charts and algorithms, aim to detect and address early signs of clinical deterioration, improving patient outcomes. Developed initially for non-obstetric use, EWS has been adapted for obstetrics to prevent maternal morbidity and mortality by monitoring vital signs [19]. Maternal EWS (M-EWS) is helpful in visualising trends, revealing “hidden” trends, facilitating shared understanding, and providing legitimacy for escalation that entails timely recognition of deterioration, good communication between teams, expedited treatment, and/or referral [20, 21]. M-EWS is feasible and holds potential in resource-limited settings, such as Nigeria, where clinical parameters comprising most M-EWS versions (such as respiratory rate, heart rate, blood pressure, temperature, oxygen saturation, and consciousness level ((using the AVPU = Alert, response to Voice, response to Pain and Unresponsive)) are routinely collected [22]. M-EWS, where utilised in Nigeria, is primarily implemented via a paper proforma. A 2019 systematic review concluded that there is limited evidence of the effectiveness of EWS in reducing maternal death across all settings, highlighting the need for more research in this area [19]. Incorporating patient-level risk factors alongside minimal clinical data in developing context-appropriate M-EWS could enhance the timely identification of maternal sepsis in settings such as Nigeria. 

### Extension to current methods - big data in maternal sepsis

Artificial Intelligence (AI) and Machine Learning (ML) have significant potential to enhance healthcare research, especially in LMICs, by reducing health inequalities and easing the burden on healthcare systems [23]. Mobile apps for monitoring patients’ vital signs and detecting early warning signs can aid frontline workers in remote areas lacking traditional medical equipment. For instance, AI has been used in India to diagnose neonatal sepsis via cloud-based platforms that support data analytics, including ML, to operate on standardised data across different regions to generate neonatal sepsis predictive scores [24]. Additionally, the Global Maternal Sepsis Study (GLOSS) provides actionable criteria for identifying maternal infection and sepsis and standardised data to improve diagnostics and treatment globally [25]. A suggested catalogue of standard data points is included in Table 1. Designing structured data inputs (essential for training AI- and ML-based systems for maternal sepsis detection and management) requires a clear understanding of the most predictive clinical and behavioural variables, as well as their routine availability and reliability.

**Table 1:** Adapted from The global maternal sepsis study and awareness campaign (GLOSS) [19]

Maternal Clinical findings
* • Temperature instability (core body temperature higher than 38.0 °C or lower than 36.0 °C) *
* • Tachycardia (heart rate greater than 110 beats/min) *
* • Tachypnoea (respiratory rate greater than 24 beats/min) *
* • O2 saturation, PaO2/FiO2 *
* • Diaphoresis *
* • Nausea or vomiting *
* • Hypotension or shock *
* • Oliguria or anuria *
* • Pain (location based on site of infection) *
* • Altered mental state (confusion, decreased alertness, Glasgow Coma Scale score) *
* • Decrease capillarity refill, clammy or mottled skin *
* • Fetal distress (fetal tachycardia, acidosis)*
Maternal Laboratory findings
* • Leucocytosis or leukopenia, immature neutrophils *
* • Positive culture from infection site or blood *
* • Hypoxemia *
* • Thrombocytopenia, INR, PTT *
* • Metabolic acidosis *
* • Hypoperfusion, increased serum lactate *
* • Low arterial pH *
* • Increased base deficit *
* • Elevated serum creatinine *
* • Elevated liver enzymes, bilirubin *
* • Serum urea *
* • Serum sodium *
* • Serum potassium *
* • Hyperglycaemia in the absence of diabetes *
* • Disseminated intravascular coagulation*

### ​​Risk factors for maternal sepsis in Nigeria

Maternal sepsis risks in LMICs are driven by factors at the health system and patient levels. Deficiencies in health systems and poor access to obstetric care in LMICs intensify the risk of maternal sepsis [26]. Poor implementation of aseptic procedures during delivery predisposes maternal patients to infections; this risk is partly driven by the incidence of home birth in the LMICs setting (reported as 59.1% in a Nigerian study [27]), attended mainly by traditional birth attendants [27, 28].

Patient-level risk factors in maternal sepsis include maternal age, unbooked status (lack of antenatal care services from formal healthcare facilities), comorbidities, and Caesarean Section (C-S) birth, among others [27, 29, 30]. Human immunodeficiency virus (HIV) infection, as a comorbidity in mothers, increases the risk of developing other infections and sepsis [29, 30]. C-S delivery can be a predictor or outcome of maternal sepsis. Antepartum sepsis increases the risk of C-S; the incidence of postpartum sepsis is higher in patients with C-S than in vaginal delivery; this is attributed to the increased risk of contamination during the procedure [31]. 

### Aim and objectives

This study aims to advance sepsis early diagnosis evidence base by assessing patient-level risk factors associated with maternal sepsis in a resource-limited setting.

Our objectives include:

1. exploring the associations between patient-level risk factors (including health-seeking behaviour) and maternal sepsis at a tertiary hospital in Nigeria;
2. assessing the difference in rates of fetal outcomes between patients who had sepsis and those who did not; and.
3. ascertaining the practicalities of obtaining the minimum dataset required to accurately detect women at risk of maternal sepsis in a busy urban Nigerian hospital.

## Methods

### Study design and setting

This retrospective study was conducted at the University College Hospital (UCH) Ibadan, a tertiary hospital in southwestern Nigeria that provides specialist care and serves as a referral centre for neighbouring and distant primary, secondary, tertiary and private health facilities. The facility gives access to various case mixes, with most patients admitted as high-risk or complex obstetric cases referred to the hospital. We extracted and digitised paper-based data from patient health records in the Obstetrics and Gynaecology Department from 2016 to 2020.

### Participants

Eligible participants were women who received care at the Obstetrics and Gynaecology unit of UCH, Ibadan, between January 1, 2016, and December 31, 2020. Inclusion criteria were: (1) women aged 15–49 years; (2) who were pregnant, in active labour, or within six weeks postpartum during the study period; and (3) who received care as either in-patients (admitted to antenatal, labour, postnatal, or gynaecology wards) or outpatients (attended antenatal, postnatal, or gynaecology clinics). Women presenting exclusively for infertility treatment, elective surgical procedures unrelated to pregnancy, or routine gynaecological screenings were excluded.

### Variables

The explanatory variables are categorised into four groups: patient sociodemographic characteristics include age group, ethnic group (Yoruba, Igbo, Hausa, and Others), and educational attainment, specifically post-secondary education status. Second, antenatal care status, as an indicator of health-seeking behaviour, is captured through the distinction between booked and unbooked status during pregnancy. Third, pre-existing medical conditions (comorbidities) include the presence or absence of respiratory conditions, sickle cell disease, hepatitis, HIV, hypertension, and peptic ulcer. Fourth, obstetric and clinical interventions/procedures are represented by caesarean section and catheter use (see Appendix 1). The variables extracted (measures of health-seeking behaviour, comorbidities, procedures, maternal sepsis, and patient characteristics) were chosen based on clinical judgement and literature review, which provided details on potential risk factors. Trained clinical research assistants manually extracted and digitised data from paper/hardcopy clinical records into an electronic database developed for this study. The data was entered into the database using a predefined proforma software. Data were standardised in the data cleaning process.

### Quality control

Before extracting and digitising the data, all research assistants involved in the process attended a mandatory 2-day training on data extraction and entry into the electronic database. Trial extraction exercises were performed during the training, with all identified issues addressed before the main process commenced. As a quality control measure, during data extraction and digitisation, two registrars from the Obstetrics and Gynaecology Department at the UCH were recruited to randomly select 25% of extracted data daily for every research assistant and verify its correctness, completeness, and accuracy. The two registrars flagged any discrepancies noticed individually. These discrepancies were reconciled among the registrars and then forwarded to the supervisors, who brought the issue to the attention of the research assistant involved. Any research assistant who failed to meet 90% accuracy was subjected to further training, supervision, and guidance. The validation exercise identified a 4% error rate, all of which originated from the data entries of a single research assistant. The low rate of errors can be attributed to the mitigation strategies implemented, including the rigorous training of data extractors with repeated hands-on demonstration exercises, and the use of built-in Excel functions, such as cell restrictions to specific data types or values, dropdown menus, and conditional formatting to flag issues like duplicate entries.

### Study size

We extracted 4,895 entries for 4,510 patients. Each entry represents a different pregnancy episode; few patients had multiple entries, but analyses were restricted to unique mothers and latest pregnancy in cases of multiple epidsodes. To examine the association between patient-level risk factors and sepsis, we analysed data for the 565 maternal patients with a record of infection.

Sepsis was diagnosed according to the obstetric-adjusted Systemic Inflammatory Response Syndrome (SIRS) criteria [32] with a score of equal or greater than two and confirmed with blood culture or cultures from any suspected source of infection. SIRS is a clinical condition characterised by a generalised inflammatory reaction that can be triggered by infection, trauma, or other insults. It is diagnosed when two or more of the following criteria are present: abnormal body temperature (> 38 °C or < 36 °C), elevated heart rate (> 90 beats per minute), increased respiratory rate (> 20 breaths per minute or PaCO₂ <32 mmHg), and abnormal white blood cell count (> 12,000/mm³, < 4,000/mm³, or > 10% immature neutrophils). In obstetric care, these thresholds may be adjusted to account for the normal physiological changes of pregnancy, such as elevated heart rate and respiratory rate, thereby reducing the risk of overdiagnosis. In the context of this study, sepsis was diagnosed using the obstetric-adjusted SIRS criteria, with a score of two or more, and it was confirmed by a positive blood culture or culture from another suspected source of infection.

### Statistical analyses

Descriptive statistical methods were used to explore the distribution of the data and the difference in rates of fetal outcomes between patients who had sepsis and those who did not. We conducted initial univariable prescreening. We then used a stepwise forward selection method to fit a multiple logistic regression model to examine the association between risk factors -health-seeking behaviour (booking status), comorbidities (respiratory conditions, hepatitis, sickle cell, HIV, hypertension, peptic ulcer) and procedures (caesarean section birth and catheter)- and maternal sepsis, adjusting for patient characteristics (age, having post-secondary education, ethnic group). Cases with missing values were excluded. Covariates significantly associated with maternal sepsis in univariable models (*p* < 0.05) were included to minimise overfitting and multicollinearity by focusing on covariates most likely to influence the outcome; however, key sociodemographic confounders—maternal age, education, and ethnic group—were retained in the multivariable model regardless of univariable significance, in line with best practice [33, 34]. Building on the model, we conducted subgroup analyses by introducing two-way interaction terms to assess whether the association between the risk factors and maternal sepsis varied by patient age, education, and ethnic group. The subgroup analyses were important to explore whether the impacts of key risk factors on maternal sepsis are consistent or differ in strength and direction across sociodemographic groups, which can help identify vulnerable populations and inform more targeted interventions. Interaction was assessed by creating multiplicative terms between the exposure and each subgroup variable and including these terms in the regression model (e.g., exposure##subgroup). Statistical significance of the interaction was evaluated using the p-value associated with the interaction term. No formal adjustments for multiple comparisons were applied. However, findings from interaction analyses were interpreted cautiously in light of potential Type I error due to multiple testing.

Inferential statistical analyses in this study were restricted to maternal outcomes, with maternal sepsis as the primary outcome of interest. While fetal outcomes were not the focus of the statistical modelling, we reported descriptive statistics to highlight potential differences in fetal outcomes between patients with and without maternal sepsis. This approach allows for clinically relevant insights while maintaining alignment with the study’s core analytical objectives. All analyses were conducted using Stata (version 17.0).

## Results

Characteristics of the 565 maternal patients with an infection are reported in Table 2. The sample was aged 14 to 57 years, with a mean age of 30.7 years (SD 5.8). Most of the participants with an infection (80.9%) identified as Yoruba ethnic group; reflecting the demographic composition of the region. Our sample was skewed with regard to educational attainment among the sampled patients compared to the general population; 47.3% (267) of those with an infection had post-secondary qualifications. About one-fifth (111) of the maternal patients with any form of an infection had sepsis. The descriptive statistics show that the proportion of those unbooked, hypertensive, had a catheter and a caesarean section was higher among the sepsis subgroup than in the general infection group.

**Table 2:** Sample characteristics

		Sepsis *n* (%)	Total infection *n* (%)
Total		111 (100)	565 (100)
Age	Less than 35 years	83 (74.8)	405 (71.7)
	35 years & above	28 (25.2)	140 (24.8)
	Missing values	0 (0)	20 (3.5)
Ethnic group	Yoruba	87 (78.4)	457 (80.9)
	Igbo	8 (7.2)	33 (5.8)
	Hausa	4 (3.6)	9 (1.6)
	Others	6 (5.5)	27 (4.8)
	Missing values	6 (5.4)	39 (6.9)
Post-secondary education	Yes	33 (29.7)	267 (47.3)
	No	33 (29.7)	134 (23.7)
	Missing values	45 (40.6)	164 (29.0)
Booked status	Booked	9 (8.1)	187 (33.1)
	Unbooked	91 (82.0)	326 (57.7)
	Missing values	11 (9.9)	52 (9.2)
Respiratory condition	Yes	1 (0.9)	8 (1.4)
	No	110 (99.1)	557 (98.6)
	Missing values	0 (0)	0 (0)
Sickle cell	Yes	1 (0.9)	10 (1.8)
	No	110 (99.1)	555 (98.2)
	Missing values	0 (0)	0 (0)
Hepatitis	Yes	1 (0.9)	8 (1.4)
	No	104 (93.7)	525 (92.9)
	Missing values	6 (5.4)	32 (5.7)
People Living with HIV	Yes	1 (0.9)	11 (1.9)
	No	110 (99.1)	554 (98.1)
	Missing values	0 (0)	0 (0)
Hypertension	Yes	4 (3.6)	17 (3.0)
	No	101 (91.0)	516 (91.3)
	Missing values	6 (5.4)	32 (5.7)
Peptic ulcer	Yes	1 (0.9)	18 (3.2)
	No	104 (93.7)	516 (91.3)
	Missing values	6 (5.4)	31 (5.5)
Caesarean section	Yes	39 (35.1)	168 (29.7)
	No	72 (64.9)	397 (70.3)
	Missing values	0 (0)	0 (0)
Catheter	Yes	45 (40.5)	173 (30.6)
	No	66 (59.5)	392 (69.4)
	Missing values	0 (0)	0 (0)

### Sepsis and fetal outcomes

We examined the distribution of fetal outcomes (livebirth, miscarriage, abortion, and stillbirth; and early neonatal death) by infection and sepsis status using descriptive statistics (see Table 3), without inferring statistical significance. Patients with sepsis had the lowest rate of livebirth (29.7%) compared to those with (41.8%) and without (82.1%) an infection. Proportions of stillbirth (intrauterine fetal death) and early neonatal deaths were highest among patients with sepsis (15.3% and 1.8%) compared to those with (13.2% and 2.1%) and without (4.5% and 1.7%) an infection. However, the rate of abortion (including miscarriage) (this group includes both spontaneous miscarriage -defined as the unintentional loss of pregnancy before 20 weeks of gestation due to natural causes- and induced abortion -intentional termination of pregnancy through medical or surgical means) was slightly higher among those with infection (3.9%) than among sepsis patients (3.6%).

**Table 3:** Fetal outcome by infection and sepsis status

Fetal outcome	Total Infection *n* (%)	Sepsis *n* (%)	No Infection *n* (%)	Total *n* (%)
Total	565 (100)	111 (100)	4329 (100)	4896 (100)
Live Birth	236 (41.8)	33 (29.7)	3554 (82.1)	3790 (77.4)
Stillbirth (Intrauterine Fetal Death)	75 (13.2)	17 (15.3)	194 (4.5)	269 (5.5)
Abortion (including Miscarriage)	22 (3.9)	4 (3.6)	15 (0.3)	37 (0.7)
Early Neonatal Death	12 (2.1)	2 (1.8)	73 (1.7)	85 (1.7)
Fetal Outcome Missing	220 (38.9)	55 (49.5)	493 (11.4)	713 (14.6)

### Association between patient-level risk factors and maternal sepsis

We investigated the association between patient-level risk factors and maternal sepsis, adjusting for patient age, ethnic group and education. The estimates from the univariable (unadjusted) and multivariable (adjusted) models are reported in Table 4.

**Table 4:** Univariable (unadjusted) and multivariable (adjusted) models

Variable		Univariable (unadjusted estimates)			Multivariable (adjusted estimates)		
		Odds ratio	95% Confidence Interval		Odds ratio	95% Confidence Interval	
			Lower	Upper		Lower	Upper
35 years and above (with less than 35 years as ref.)		0.97	0.60	1.57	1.50	0.80	2.81
Ethnic group (Yoruba as ref.)	Yoruba	-	-	-	-	-	-
	Igbo	1.36	0.59	3.12	1.79	0.59	5.42
	Hausa	3.40	0.89	12.93	2.95	0.53	16.41
	Others	1.21	0.48	3.10	1.00	0.25	4.06
Post-secondary education		0.43	0.25	0.74	1.39	0.38	1.34
Booking status		0.13	0.06	0.27	0.17	0.08	0.38
Catheter		1.63	1.06	2.51	2.60	1.35	5.01
Respiratory conditions		0.55	0.07	4.55	D	D	D
Hepatitis		0.55	0.07	4.55	D	D	D
Sickle cell		0.43	0.05	3.42	D	D	D
HIV		0.38	0.05	3.04	D	D	D
Hypertension		1.21	0.39	3.79	D	D	D
Peptic ulcer		0.22	0.03	1.69	D	D	D
Caesarean section birth		1.28	0.82	1.99	D	D	D

D: Dropped from the final multivariable model as the univariable estimate was not statistically significant

The multivariable model included risk factors significant in the univariable models. Only antenatal care booking status and the use of catheters were significantly associated with maternal sepsis in the adjusted model. Maternal patients who had antenatal care services from formal healthcare facilities (booked status) were 83% less likely to develop sepsis compared to those who did not (unbooked status) (OR: 0.17; 95% CI: 0.08–0.38). In addition, having a catheter (invasive devices) increased the likelihood of maternal sepsis by more than twofold compared to not having a catheter (OR: 2.60; 95% CI: 1.35–5.01).

We expanded the models by introducing two-way interaction terms for subgroup analysis (specifically testing whether the associations between antenatal booking status and catheter use with maternal sepsis varied by age, education, or ethnic group). None of the interaction terms were statistically significant, suggesting that the effects of these risk factors were consistent across the sociodemographic subgroups in our study.

## Discussion

The study assessed patient-level risk factors associated with maternal sepsis at a tertiary hospital in Nigeria. Identifying risk factors and detecting early onset maternal infection and sepsis before it becomes severe is critical to reducing its associated maternal and fetal morbidities and mortalities, especially in resource-limited settings like Nigeria, with high maternal infection and sepsis burden [27, 29, 30]. One of the main findings from our study was that mothers who utilised antenatal care services from formal healthcare facilities (booked status) were 82% less likely to develop sepsis. Also, mothers with urethral catheterisation in situ had higher chances of developing maternal sepsis.

One of the strengths of our investigation is that we used a large patient-level dataset from a busy urban hospital in a resource-limited setting with a high risk of sepsis to investigate maternal sepsis risk factors. However, the transferability and generalisability of our findings are limited as participants were from a tertiary hospital that provides emergency obstetric care and is more likely to see high-risk and severely ill patients. As such, the experiences of the patients may not represent those of the general population of women who are pregnant, in labour or postpartum in Nigeria. Also, the recruitment of participants from one site introduces selection bias, as the sample may not reflect the broader diversity of demographic, socioeconomic, cultural, and healthcare factors present in the wider Nigerian population, thereby limiting the generalisability of the findings to other populations or settings. However, the study is set in a hospital that attracts patients with diverse characteristics with regard to socioeconomic status, type of location (urban, rural, suburban), and comorbidities.

Although diverse, our sample has a skewed distribution of educational attainment, with 66.6% of participants (267) having post-secondary qualifications. This overrepresentation reflects educational inequalities in utilising formal healthcare facilities in Nigeria, as reported in other studies on maternal health from this setting [35–37]. Higher education in this context is linked to better health literacy, greater use of formal healthcare, and improved maternal and fetal outcomes [1, 38]; thus, the skewness in educational attainment likely underrepresents the true burden of adverse outcomes such as maternal sepsis and miscarriage, abortion, or stillbirth among women with low education, who face greater barriers to care.

Another limitation of this study is precisely identifying women with sepsis, despite a 2017 WHO consensus on a definition for maternal sepsis [39]. The lack of validation studies among international populations, in addition to variations in infection types and diagnostic criteria, hinders wider uptake of this definition. This limitation has significant implications for diagnosis and clinical management, increasing the risk of misdiagnosis, inadequate treatment, or delayed care, especially in low- and middle-income countries with limited resources [39]. For instance, a woman presenting with postpartum fever, elevated heart rate, and uterine tenderness may be diagnosed with a localised infection, such as endometritis, in one setting, while in another, the same symptoms could be classified as maternal sepsis under broader clinical criteria. In this study, the lack of consensus on maternal sepsis definition challenged our ability to determine if all patients with sepsis included in the patient notes truly had sepsis.

Incorrect entry is a possible limitation in our study, given the manual transformation of data from handwritten patient notes to an electronic format. Also, for missing data, clinical observations may have been left blank when absent (i.e., a true zero value); this can limit the distinction of true missing values from true zero values, complicating the investigation of missingness mechanisms in the dataset. To reduce the potential bias from these, registrars from the Obstetrics and Gynecology Department reviewed samples of data entered.

Maternal sepsis prevalence in our study (2.27% of the 4895 episodes) compares to the rate from another study in Nigeria (0.8%) [27]. The higher prevalence observed in our study may be attributed to contextual factors (including its setting in a leading tertiary hospital affiliated with a research-intensive university that primarily manages more severe cases, and its urban location near major cities like Lagos), that likely contribute to increased case volume, greater case severity, and improved diagnostic accuracy, which together explain differences from previous Nigerian studies. Also, higher rates have been reported in other studies in Nigeria (9.34%) [40] and Tanzania (11.5%) [41].

Similar to our findings on booking status, Ouonuju et al. [40] reported a higher risk of maternal sepsis in those unbooked. Antenatal care is essential for preventing and early identifying pregnancy anomaly conditions and maternal and/or fetal morbidity/mortality. Socioeconomic factors are key predictors of the uptake of antenatal care services in this region [42] and sepsis, as seen in our study. Multiple barriers to accessing maternal healthcare, such as financial constraints, distance to facilities, limited transportation, lack of awareness about antenatal services, misalignment between antenatal care provision and the social and cultural context, and distrust in formal healthcare systems, have been reported to contribute to delayed or no antenatal booking among pregnant women in LMICs [43–45].

Socioeconomic factors, such as educational attainment, are well-established predictors of antenatal care service uptake in this region [42], which in turn influences maternal health outcomes, including sepsis risk. In our study, while socioeconomic factors showed a significant association with maternal sepsis in the unadjusted model, this significance did not persist after adjusting for other covariates in the multivariable model. This suggests that the effect of socioeconomic factors on maternal sepsis may be mediated or expressed through other patient-level factors, underscoring the complexity of interactions influencing maternal health outcomes. Education as a socioeconomic factor increases access to information, enhancing patients’ health-seeking behaviour [46]. Women with higher education are more likely to book antenatal care, adhere to medical advice, and access health services promptly, all of which are protective against sepsis. When these factors are accounted for, the independent effect of education diminishes, suggesting that its influence on sepsis risk operates indirectly through these more proximal determinants of maternal health. Higher odds of stillbirth in Nigeria have been associated with low levels of maternal education, distance to travel from home to hospital, living in a shack, maternal hypertension and previous stillbirth [47]. Although we hypothesised that the association between booking status and sepsis might be explained by socioeconomic measures such as education, our subgroup analysis did not show a significant difference by education. This absence of subgroup effects suggests that universal, system-wide interventions to reduce maternal sepsis, such as promoting early antenatal care and improving infection control, may be broadly effective across sociodemographic groups.

A key finding of our study is the increased risk of maternal sepsis in patients with urethral catheters. This aligns with the established evidence on the role of catheters as a main source of bacteremia and sepsis in hospital patients [48]. The use of an indwelling catheter predisposes to bacteriuria and urinary tract infections. A UK study showed that women’s anatomical factors, such as the shorter distance from the anus to the urethral opening and shorter urethral length with a vaginal or perineal microenvironment, may facilitate colonisation of the urethra and thus an indwelling catheter by uropathogens [49]. Aseptic urethral catheterisation, prompt discontinuation of catheters when no longer needed, and the use of antibacterial catheters when available will reduce sepsis risk in catheterised maternal patients. These practices can be promoted in LMIC settings through targeted clinical training, integration of catheter care protocols into maternal health guidelines, routine supervision, and ensuring the availability of essential supplies.

Our descriptive results showed a higher prevalence of miscarriage, fetal death and early neonatal death among women with infection and sepsis than those without an infection. Maternal sepsis is associated with increased adverse outcomes in pregnancy, such as early neonatal sepsis, neonatal deaths, fetal death and miscarriage [1, 3].

Our findings are important to advancing clinical practice and public health policies towards managing and controlling maternal sepsis in Nigeria. The results suggest that improving access to antenatal care services for pregnant women will substantially reduce the risk of maternal sepsis in the Nigerian population, and re-iterates calls for a multi-omic and health care data science approach to improve diagnostic accuracy of clinical EWS [50]. In addition, guidelines for maternal sepsis management should consider the increased risk for subgroups of patients, such as those with urethral catheters.

One of our broader objectives to be addressed beyond this study is determining the minimum dataset required to accurately detect women at risk of maternal sepsis in a resource-limited setting. Collecting this information in a busy urban hospital in Nigeria is certainly practical, and detailed analysis is possible with careful planning. However, further studies with multiple sites and sepsis records based on a more precise case definition are needed to accurately determine the minimum dataset required to detect women at risk of maternal sepsis.

## Conclusion

In this study, a high occurrence of maternal sepsis was significantly associated with patient-level factors, especially non-booking status and indwelling urethra catheter. There is a need to increase awareness about the importance of antenatal care service booking, especially in early pregnancy. The continuous collection of more detailed socio-demographic and clinical information on maternal patients in hospitals is warranted for developing prompt and specific decision-making in reducing sepsis-related maternal and neonatal morbidity and mortality rates in LMIC settings. Future efforts should prioritise interventions to improve data standardisation and surveillance for maternal health, particularly sepsis, to enhance the accuracy and comparability of outcomes across settings; such improvements can also support the effective implementation of maternal early warning systems by providing reliable baseline data and monitoring indicators.

## Data Availability

The data that support the findings of this study are available from the University College Hospital, Ibadan but restrictions apply to the availability of these data, which were used under license for the current study, and so are not publicly available. Data are however available from the authors upon reasonable request and with permission of the University College Ibadan, Nigeria. Please contact the corresponding author (Dr Philip Anyanwu philip.anyanwu@warwick.ac.uk) for more information regarding data availability and access.
